# How End-of-Life Blogs Re-Affirm the “Power to be Oneself”

**DOI:** 10.3389/fsoc.2021.775279

**Published:** 2022-01-14

**Authors:** Gill Green

**Affiliations:** School of Health and Social Care, University of Essex, Colchester, United Kingdom

**Keywords:** power, end-of-life, blogs, identity, personal growth, empowerment

## Abstract

Powerlessness generally denotes loss of control and may be experienced among those with a terminal diagnosis and, as such, empowerment is a dominant discourse in end-of-life policy in the western Anglo-Saxon world. This paper analyzes thematically blogs authored by three people with a terminal diagnosis to examine the “power to be oneself,” a concept which was identified in the “Ethics of Powerlessness” project conducted in the UK. The analysis demonstrates that the bloggers assert the “power to be themselves” which is expressed in three principal ways. Firstly, through assertion of agency to promote self-affirmation and control. Secondly, through claiming a “moral authority” expressed by providing advice not just on illness and death but also on how life should be lived. Thirdly, through discussing ideas about the future and creating a legacy. The blogs are a mechanism used to express and reinforce self-identity and to carve out a “sacred space” between life and death to nurture personal change and to project this onto a public arena. This analysis demonstrates the key role patient empowerment plays in constructing an identity with a terminal diagnosis, an element that is often overlooked in end-of-life policy.

## Introduction

A diagnosis of a long-standing condition can assault both one’s physical and ontological self (Corbin and Strauss, 1987; Lawton, 2003; Pierret, 2003) resulting in disruption of identity (Bury, 1982) or even a loss of self (Charmaz, 1983). Disruption to identity and feeling powerless are likely to be particularly pronounced among those diagnosed with a terminal illness as they have to cope with a future of declining health, dependency on others for care, inability to perform many tasks associated with “normal” life (e.g. caring for children, work) and ultimately the end-of-life. Powerlessness is not a universal reaction to a terminal diagnosis as some may feel ready to die and largely in control of their death, but empowerment and patient choice are key features in current UK guidance on end-of-life care (Borgstrom and Walter, 2015; Department of Health, 2015). Empowerment to make decisions relating to physical comfort including being pain free, perceived quality of care, and the place and medicalization of death are reported as key to end-of-life well-being in the Anglo-Saxon western world (Carr and Luth, 2019).

The discourse of patient empowerment and choice has been influential in shaping government end-of-life policy (Department of Health 2015). Empowerment, in this context is often understood to be about patients having control to make choices and determine their healthcare provision. However, promoting “empowerment” has been critiqued from both theoretical and practical perspectives. Salmon and Hall (2004) note that the ideal of empowerment as control is inadequately evidence based. They also suggest that placing responsibility with the patient rather than the clinician may serve the interests of clinicians (Salmon and Hall, 2003) and unwittingly oppose patients’ interests (Salmon and Hall 2004). An ‘Ethics of Powerlessness project (https://www.essex.ac.uk/research-projects/ethics-of-powerlessness) found that transferring responsibilities to patients can be disempowering. For example, the encouragement to remain positive, live as normal a life as possible and adopt a fighting spirit, which is common in much of the end-of-life empowerment rhetoric, may suppress expressions of emotional distress, which may have a negative impact on patients and may be experienced as disempowering.

According to Borgstrom (2015), in England patient choice among people nearing the end-of-life is often little more than a tick box bureaucratic exercise which serves to sustain the political neoliberal agenda and there is no guarantee patients will be able to action their choices, e.g. If they would like hospice care but a bed is not available. Bailey et al. (2020) also note that patient choice is constrained by resources and competing priorities of patients, professionals and institutions, and the outcome represents a struggle between the institutional logics of patient choice, professional authority and finance. In this struggle, patient empowerment and their preferences are often given lower priority than professional choices and resource constraints. In such a scenario an inability for empowered patients to realize their choices may result in heightened feelings of disempowerment.

Powerlessness emerges as a key feature in studies of terminal illness. In Sand et al. (2008) study of 103 patients from four palliative care units in Sweden, 16% stated that they had experienced powerlessness and helplessness several times per week, 49% stated that they had done so occasionally and only 35% reported no such experiences. Powerlessness and helplessness are also reported to be key issues for close family with one-third of family members of cancer patients in advanced palliative home care reporting experiencing powerlessness or hopelessness several times a week (Milberg et al., 2004). Aujoulat et al. (2007) study based on interviews about the experience of powerlessness among 40 patients with chronic conditions found that powerlessness extends well beyond medical and treatment-related issues, and that study participants all expressed distressing feelings of insecurity and a threat to their social and personal identities.

A diagnosis of a terminal illness can unleash a profound sense of insecurity and disempowerment (Aujoulat et al., 2007). In response to this insecurity and loss of control, Aujoulat et al. (2008) study of patient empowerment identifies a double process of ‘Holding on’ to one’s previous pre-illness self to establish a sense of control and ‘Letting go’ to establish a sense of coherence and meaning in the knowledge that not everything can be controlled. This resonates with the existential ambivalence identified by Flaherty (2018) in her analysis of one woman’s transitory process following a terminal diagnosis which involved a focus on both dying and living well. Others have also noted how people approaching the end-of-life periodically foreground and background living and dying selfhoods (Lowrie et al., 2019).

Wakefield et al. (2018) conducted a systematic review of patient empowerment among those with a life limiting illness at an advanced stage. They note that the empowerment paradigm is different among people with a terminal diagnosis compared to those with a long-term condition. Whereas self-management and self-efficacy are key features with the majority of patient empowerment constructs based on people with long term conditions, their interpretive synthesis of the literature of empowerment among people with a terminal diagnosis identifies other concepts of empowerment. Of particular note in their conceptual model of patient empowerment for adults with advanced life-limiting illness is that self-identity is at the core. Identity is an aspect that is overlooked in many end-of-life policies.

Powerlessness and identity were key concerns of the “Ethics of Powerlessness” project, which explored the ethical challenges that arise from human experiences of powerlessness, especially in contexts of end-of-life care. The project aimed to develop a framework to assist understanding of how individuals respond to situations of diminished agency, from the perspective of affected individuals and those supporting or caring for them. The project examined the meaning of power starting from the two types of power identified by Allen (1998), “power to” achieve some goal and ‘power over’ to make someone do something that they would not otherwise have done. They speculated that “individuals experience powerlessness when the lack of power-over and/or power-to results in a lack of power to be” (Batho, 2015 p28). They labeled this third dimension of power as the ‘power to be oneself’ noting that a lack of ‘power to be oneself’ denotes an inability to do the things you used to do and be the person you used to be, leaving “one feeling as though one does not know who one is anymore” (Batho, 2015, p.28).). This is linked to a loss of familiarity with one’s environment, increased insecurity and existential loneliness (Batho, 2016). Batho notes that a terminal illness may thus impact but not necessarily negate entirely the “power to be oneself,” which builds on Aujoulat et al. (2008) notion that some people are able to become a “same but different person” (p1236) and like Wakefield et al. (2018) places self-identity at the centre.

This paper examines power, identity and the concept of “power to be oneself” through the analysis of blogs authored by people who are faced with a terminal diagnosis. The concept of the “power to be oneself” was the driver of this paper and the empowerment concept aligns with current end-of-life policy debates, hence the focus on power in this paper rather than alternative conceptual models such as meaning-making. Meaning-making has been identified as a key motivation for blogging about challenging topics as in an analysis of blogs about sexual assault, which helped the bloggers understand and recover from the abuse and achieve positive growth (Fawcett and Shrestha, 2016). Whilst the conceptual model in this paper is oriented to power, meaning-making is clearly discernible in the blogs and is sometimes evident in the analysis discussed below. This paper seeks to understand to what extent is the “power to be oneself” evident in end-of-life blogs, how is it expressed and what is its function? How do expressions of highly personal end-of-life journeys in semi-public spaces on-line inform our understanding of empowerment and identity associated with end-of-life?

## Methods

### Selection of End-of-Life Blogs

Those approaching the end-of-life face the ultimate ending of self, hence my decision to focus on this time of life. Whilst there are a number of studies exploring powerlessness among people with long-term illness (e.g. Margaretha Strandmark, 2004)) and near the end-of-life (e.g. Lawton, 2000), the use of secondary data accessible on-line is relatively under-explored. Moreover, blogs produce naturalistic data (Seale et al., 2010) and the author decides what to include and exclude. That said, blogs are of course performative and written with an audience in mind, albeit one that is public and mainly anonymous, and therefore the blog content is likely to be influenced by the bloggers assumptions about who may read it. Nevertheless, it is the blogger not the researcher that determines the content of the blog and it is written in their own words and, unlike published work, there is no independent editorial hand.

Defining “end-of-life” or “terminal” is not straightforward as many people who have received such a diagnosis may not choose to define it as such. Furthermore, adjuvant and palliative treatment may be delivered concurrently making definition of terminal more difficult to define. I therefore focused on blogs from individuals who stated that they had received a terminal diagnosis and had been told that their life span was likely to be less than five years (generally less than two).

Google searches were conducted in July 2018 using key words “blogs” and “end-of-life”/“terminal illness”/“death”/“dying.” I excluded blogs from: organizations rather than individuals; family members; health professionals. Following these exclusions, I identified 32 blogs and I contacted all those who had been active within the last year and where there was an obvious point of contact for a personal message.

### Ethics

There are ethical challenges related to research with people approaching the end-of-life, including using their blogs as source material (see the Association of Internet Researchers, 2019; UKRI Economic and Social Research Council, 2020). Whilst bloggers can be both subject and author and the material is publicly available, often inviting contact and comments from readers, researchers are unlikely to be the bloggers’ target audience (Sixsmith and Murray, 2001). Furthermore, blogs are not anonymous unless the blogger uses a pseudonym. Blogs are public but this does not necessarily mean that they are ‘fair game’ for researchers (Eastham, 2011). There is also an unequal power relationship between the researcher who knows intimate details about the blogger, and the blogger who knows nothing about the researcher (Snee, 2013). The potential participants were at a vulnerable time of their lives and even seeking informed consent was not straightforward as bloggers may find a request to participate in research to be intrusive. Furthermore, it was not always possible to send a private message to the blogger or, for the bloggers who had died, to find an appropriate significant other to message to seek their consent.

Once ethical approval from a university ethics committee (Reference number 15025) had been received, a private message was sent to the blogger or their significant other (in case of death) electronically providing details of the study and asking their consent to use the blog in the analysis. This included a brief information sheet and consent form. The consent procedure sought authorization: to use quotes from the blog; to name the blogger; to provide a live link to the blog. Four of the seven contacted replied to say they were interested but one did not return the consent form and therefore was not included in the analysis.

### Analysis

I began the analysis by reading a selection from all 32 blogs (written by 13 male and 19 female bloggers) that I identified to get a feel for the content. I then conducted extensive analysis of the three blogs selected for in-depth analysis and identified core themes (described below). I then returned to the remaining 29 blogs to check that the core themes were reflected among the wider terminal illness blogger community and were applicable to a broader demographic and in different cultural contexts. These additional blogs were thus used for quality assurance. As I only had consent from the three selected bloggers to use the material in their blogs for the analysis, and as bloggers are readily identifiable if quotes from blogs are used, I do not include any verbatim material from the additional 29 end-of-life blogs in this paper.

Indicative Thematic Analysis was undertaken on the three blogs selected for intensive examination in accordance with Braun and Clarke (2006), Braun and Clarke (2012) approach. A theme is defined as “a patterned response or meaning within the data set” (Braun and Clarke, 2006, p. 11). My aim was to generate salient themes related to end-of-life and to this end I excluded much of the material about treatment and symptoms which spoke more about the struggles of staying alive rather than contemplating end-of-life. Text from the blogs were loaded onto MAXQDA a software package to assist the analysis of qualitative data. First order codes were identified and then grouped together to generate initial theme titles. I then examined the meaning of each theme and linkages between them to further refine/collapse them to identify core themes concerning empowerment related to end-of-life.

I was reflexive throughout this process in recognition that the researcher is part of the research (Finlay, 1998) and that I needed to be aware of how my own beliefs and judgements were shaping the research process. This was particularly pertinent in this research as while conducting the analysis I was supporting a close family relative to cope with a terminal diagnosis. She had no desire to write about her end-of-life experience or publicize her journey through social media and this therefore shaped my views about why some people choose to express highly personal matters in a semi-public arena while others do not. In many ways this disjuncture between the bloggers response and that which I experienced in my personal life, acted as a useful counterpoint in my interpretation of the data. It helped me to reflect on the different ways in which power and control can be exercised by people with a terminal diagnosis and also acted as a yardstick to reflect upon whether the core themes identified in the blogs were specific to bloggers or could be applicable to a wider group of people with a terminal illness.

## Results

### The Bloggers

In order to place the quotes and the data within a wider context, it is important to provide brief details about the three bloggers and blogs used in the analysis.

Susan was diagnosed with an aggressive terminal cancer in March 2017 aged 51 and died in August 2018. She was a teacher who lived with her partner in Canada. Shortly after her diagnosis she began her blog called The Death Project. She stated that her main motivation for blogging was to challenge what she saw as portrayal of death as taboo and something to fear. She wrote 51 blogs in total, the last just a month before her death.

Don was diagnosed with cancer in 2007 and was told it was terminal in January 2018 when he was aged 56. He decided to pursue medical assistance in dying and blog about his journey on Dying With Dignity Canada website. Don’s blog focuses upon his journey with medically assisted dying (MAID) which become legal in Canada in 2016. He wrote 6 blogs between January and April 2018 when he died a medically assisted death. The main focus of the blogs is the process of assisted dying and the hoops that need to be navigated.

Sophie Sabbage, aka the Cancer Whisperer, was diagnosed in 2014 age 48 with lung cancer with multiple metastases. Despite a prognosis of less than a year to live, she continued to live until her death in October 2021. She has authored two books “The Cancer Whisperer” (Sabbage, 2016) and “Life Shocks” (Sabbage, 2018). She was a high-profile patient activist committed to changing the “battle” narrative about cancer and describes herself as a “Stage four cancer thriver and global authority on how to thrive in adversity.” In 2019 she received the 2019 Global Lung Cancer Alliance award for “Excellence in Journalism.” She wrote 55 blogs from time of diagnosis (late 2014) to February 2021 with 39 of them written in the year following diagnosis.

### Identifying Core Themes

Having identified the themes from the three blogs that were related to end-of-life and powerlessness/empowerment, I further refined these to locate stable core themes to emerge from the blogs. I checked that the core themes from the three blogs selected for analysis were present in the other 29 terminal illness blogs that I identified and found that they were.

#### Core Themes

**Table:** 

Initial theme	Final core theme
Feeling loved	Identity: self-affirmation and control
Cherishing life and normal life pleasures	
Motivation to blog	
Acceptance, love and hope whilst acknowledging sadness	
Planning death and dying	
Philosophy of life and life tips	An authoritative voice
Thoughts about death and dying	
Thoughts about symptoms and treatment and a terminal diagnosis	
How to support people with a terminal diagnosis	Legacy
Commitment to cause, e.g. assisted dying	
Motivation to blog	
Caregivers and loved ones	

### Identity: Self-Affirmation and Control

Narratives that challenged the assault on identity that is associated with diagnosis of a terminal illness were prominent in the blogs. These were expressed by re-affirming selfhood and taking conscious steps to take control of one’s life and, in Don’s case, his death.

Acknowledgement that one has a terminal illness is a pre-requisite for writing an end-of-life blog and narratives of acceptance of having a terminal diagnosis dominate. For example, in Susan’s first blog she writes:

I have been diagnosed with an aggressive terminal cancer, stage four uterine leiomyosarcoma. It is not curable and doesn’t respond much to treatment… I especially want those who are concerned about me to know that I am at peace. This is not the kind of cancer that one hopes to beat. I have found, in fact, that simple acceptance is easiest. Acceptance has also allowed me to share incredibly special moments with my loved ones. (Susan “Sharing my news” 31.3.2017).

In Susan’s blogs, the language of acceptance is used to establish a foundation from which she can cherish life and find purpose: “I can hardly imagine feeling more blessed or more loved than I do now. I often feel that I am in state of grace. I am grateful for each day. I live with purpose and joy.” (Susan “On Control” 15.5.2017). This sense of purpose is a key motivation for her blog “The Death Project.” She reports that the blog is a mechanism she uses to continue to find meaning in life, thus reinforcing her identity:

I see from this that dying really means living. This death project requires that I keep living in the way that I have always found meaningful until I simply run out of time. (Susan “On work” 13.5.2017).

This affirmation of selfhood is also evident in many of Sophie’s blogs which express narratives re-affirming the self in response to the assault of a terminal illness diagnosis. In “My beautiful brain” (21.04.2015), she describes her journey from her “darkest hour” of being told that she had multiple metastases in her brain to a brain scan report received 5 months later, which described an “excellent response to treatment” and “No metastases detected.” She writes:

They couldn’t count the tumours. They wanted to radiate my whole brain. I was in grave danger of losing myself, which was more frightening than losing my life.
But here I stand.
On solid ground.
Living with cancer.
Lucid. Grateful. Awake.
Full of wonder.
And so I walk. (Sophie).

Another way in which identity was reinforced by all three bloggers was by them reportedly taking as much control of their lives as they were able to. For Sophie, taking control was manifest in her active treatment-seeking which included alternative as well as mainstream therapies. It is clear in her blogs that this helped her to assert autonomy, and that this was an important part of her identity:

I am proactively engaging in my own treatment of my own disease, which shifts me from victim of my illness to author of my wellness—however long that may last. This does not mean I am deluded or denying the gravity of my situation. It just means I am still in charge of my life (Sophie “Myth of False Hope: power comes from engaging in my own treatment” 07.03.15).

Don, however, provides the clearest example of taking control. He was committed to the cause of MAID prior to his terminal diagnosis and, as he became increasingly unwell, and less able to take part in social activities, he decided to take active steps to pursue it:

I have been very unhappy with my life recently, particularly as my voice failed me, which made conversations nearly impossible. My request for MAID is imminent as I don’t want to exist in relative isolation for any extended period. (Part 2 of Don’s journey “My plans to access medical assistance in dying” 2.3.2018).

His blogs demonstrate how he controls his death ensuring that it takes place with the people he loves around him and with his favorite track Deep Purple’s “Child in Time” playing.

A date has been selected. I will end my life in the late morning of April 20th at home accompanied by my wife as well as my mother and two sisters. My sisters are flying in from the United States and will remain local for several days to support [my wife] and my mom. (Part 7 of Don’s journey “This is Don… signing off” 9.4.2018).

According to his family, “Don left this world exactly as he wanted to go… he slipped away quietly, peacefully, and on his own terms.” In this sense, he was in control.

### Asserting an Authoritative Voice

A core and stable theme that was evident in the selected blogs is that of the “authoritative voice.” This “voice” was a feature in many of the blogs and offered advice about illness, death and life itself and how it should be lived.

### An Authoritative Voice—About Illness

Drawing upon their experiential expertise, the bloggers claim to have acquired transferable knowledge about living with a terminal illness. For example, as evidenced by the quotes below, Sophie indicates that the experience of living through her own journey has conferred on her the ability (or even the right) to give advice to others about the management of illness:

I’m not interested in making political arguments or qualified to do so. But, as a patient with a terminal cancer diagnosis, I am qualified to talk about my desperation, my vulnerability and my hope. (Sophie “Myth of False Hope” 07.03.2015).

Sophie includes many tips about how to approach illness and also, in response to her experience of receiving well-meaning but unhelpful remarks from friends and acquaintances, advice about how to avoid this:

So instead of expecting them to know how to support me I wrote them an email with a list of “what helps” and “what doesn’t”…. don’t wait for them to figure it out. Tell them. (Sophie “How can I help” 14.03.2015).

### An Authoritative Voice–About Death

Having experience of receiving a terminal diagnosis and with the end-of-life approaching an authoritative voice was also evident when they blogged about death. Sophie offers advice about how to access a “sacred space” after receiving such a diagnosis in which you can see clearly “what you need to do”:

… when a doctor tells you that your illness is incurable and, worse, puts a timeline on your demise. When this happens there is a sacred space to step into where you can see clearly and know with confidence, even certainty, how you want to be and what you need to do. (Sophie “Nocebo Effect” 20.10.2015)

Don uses his terminal status to speak with authority aligning his political views about, and personal preference for, medical assistance in dying.

I have no intention of subjecting myself to unnecessary pain and suffering when I die. We euthanize our pets humanely, but it is only recently that we have started applying humane dying to our own species. I want medical assistance to ease my way out of this life—nay, I demand it! (Part 2 of Don’s journey “My plans to access medical assistance in dying” 2/3/2018).

His authoritative assertion that “I demand it!” suggests that he sees this as his right.

### An Authoritative Voice—About Life

Whilst bloggers with a terminal diagnosis can justifiably claim to have experiential insight into both illness and death, what was also sometimes apparent in the blogs was a desire to give advice about life in general and how it should be lived. This was a regular feature of both Sophie’s and Susan’s blogs. Sophie claims that, drawing on her own illness experience and the positive feedback from people reading her blogs, she is well-placed to give advice about how to deal with adversity, saying: “I am in the business of awakening the human spirit: of enabling people to unleash their courage and creativity in response to the hand Life deals them.” (Sophie ‘Never say die’ 31.3.2015). Susan also offers her readers life advice asking them to do “good things” and free ourselves from “self-doubt”:

Do all those good things that make the world a more beautiful, worthwhile place to live. This is our great gift as humans, one we can all participate in or at least facilitate or enjoy. And please, most especially, don’t let yourself be paralyzed by self-doubt and fear of failure. (Susan “On creativity” 22.5.2017).

She also urges us to think not only of ourselves but to “make a contribution to society,” saying, “If you have the privilege of choosing a career, choose the path that allows you to offer your gifts as a contribution to society.” (Susan “On giving gifts” 23.8.2017).

### Legacy

The third core theme was the desire to leave a legacy, which appears to have been an important motivation for all three bloggers. This was illustrated in a number of ways. Susan for example talks about setting up a special fund and Sophie records video diaries of her journey to “create a YouTube channel that might help others who walk this paradoxical path” (“Pre-scan jitters” 25.3.2015). For Don, evidence of legacy was largely a political expression to support the movement advocating MAID and his “journey” forms part of the Dying With Dignity Canada website, a national human-rights charity committed to improving quality of dying. He writes:

Now that my end is rapidly approaching, I want to share with you my journey from diagnosis to death and my engagement with the group responsible for MAID here at The Ottawa Hospital. Over the coming months, I hope to share this as the events unfold and hopefully it will be useful to help guide others in their own journey. (Part 2 of Don’s journey “My plans to access medical assistance in dying” 2.3.2018).

Likewise, Susan clearly states that a key motivation for “The Death Project” was to share her journey in order to challenge what she perceived to be a taboo about talking about end-of-life. She talks about her blog being a mechanism to promote social change about attitudes to death:

The immediate and overwhelming feedback to my writing showed me that here was another area in need of profound social change. …So that became the new focus of my social-change work: talking openly about dying to counteract our cultural denial of death. (Susan “On changing the world (1)” 28.1.2018)

The motivation to share her story in order to “make a difference” is a feature in many of Sophie’s blogs as well as her books and on-line courses she runs. She thanks those who have responded to her blog noting:

[it is] humbling to know that I have expressed something of your experience through my own. When “my story” becomes “our story” I know I’ve been of service to something bigger than my own desire to heal, a grander narrative about a disease that wreaks havoc on the lives of millions, yet calls us into an ever-deepening relationship with ourselves and That Which Made Us. (Sophie “Response to comments” 27.3.2015).

## Discussion

Previous empirical and theoretical work has noted the strong associations between the social processes involved in dying and the wide variations in socioeconomic and cultural contexts found in human populations (Glaser and Strauss, 1965; Lawton, 2000). The increasing pervasiveness of social media has inevitably had an impact on the experience of living with a terminal diagnosis and the end-of-life experience and this analysis offers insight into how blogs can enhance one’s self-identity at this time. Whilst the three bloggers discussed here may have found other avenues to create a space for personal growth and development had they not written blogs, there is evidence that they used the blogs as a vehicle to reassert agency when faced with a terminal diagnosis. The blogs thus challenge the powerlessness that Sand et al. (2008) note is reported by many approaching end-of-life. The blogs are used to reinforce the bloggers’ identity through expressing their selfhood and autonomy. They assertively express to all their readers (both known and unknown) who they are, how they want to live life with a terminal diagnosis and, for Don, how he wants to die. In this sense, they are a clear expression of the “power to be oneself” and are a vehicle through which the profound change engendered by a diagnosis of terminal illness is used to accentuate this power. “Power to be oneself” is also expressed by a claim to having “moral authority” based on experience, closeness to death and limited life. The bloggers therefore felt both entitled and obliged to give opinions about certain life and death matters. This aspect is only rarely discussed in the literature about illness and death.

Terminal illness bloggers seem to exist in a liminal state between life and death, related to the process of foregrounding and backgrounding life and death noted by Lowrie et al. (2019), which gives them license to claim a special spiritual status. For example, Sophie calls herself “the cancer whisperer,” which suggests that like “horse-whisperers” who have a special bond and power to control wayward horses, she has a special bond with others faced with a terminal diagnosis. It also suggests a special bond to the illness itself and a skill in dealing with it. Having acquired and exercised that skill it has become part of what it means to be herself (and of her power to be herself).

The blogs clearly reclaim the bloggers sense of self albeit a self that the bloggers acknowledge is somewhat changed by the experience of having a terminal illness. In this way, blogs can be seen as an expressive mechanism through which people with a terminal diagnosis are attempting to reassert their agency, autonomy and power to be themselves. They thus play an important role in protecting and promoting identity which, according to Wakefield et al., lies at the heart of empowerment among those nearing end-of-life.

“Power to be oneself” is further advanced by the desire seen in all three blogs to leave a legacy so that their life and death has meaning. Whilst legacy projects been found to be helpful for those facing the end-of-life (Allen et al., 2008) and are a regular part of hospice care (Vidal et al., 2018), when viewed through the lens of power, one sees the ‘power to be oneself’ in a future world after one’s demise, is projected onto a public stage. Blogs are written to an audience. Some indeed begin in response to concerned enquiries from friends and families, but they are publicly accessible and written also for an unknown audience, some of whom provide feedback on the blog site. This enables the blogger to talk to a much wider audience than those who do not engage with social media to talk about their impending death and this appears to strengthen the bloggers power to be themselves and give them authority to provide advice to others. Indeed, many of the blogs explicitly say that a key motivation is to help others who are faced with a similar diagnosis. This type of authority and bequest, which is rooted in the personal experience of terminal illness, has been conceptualized as “ethical capital” (Williams et al., 2010).

The analysis thus illustrates the key role that patient empowerment plays in constructing an identity with a terminal diagnosis. The bloggers all demonstrate a desire to determine their treatment and how to live but beyond this, the blogs represent part of their identity-work to be themselves and to counter feelings of inferiority and powerlesseness engendered by a terminal diagnosis. In response to threats to self-hood as detailed by Charmaz (1983), the analysis of the blogs reveals the micro social processes as described by Scott (2015) through which powerful identities of living with a terminal illness are constructed, negotiated and maintained on social media. Whilst these micro processes have been deconstructed in a variety of settings and groups (e.g. Roschelle and Kaufman 2004; Green et al., 2006; Fine, 2012), due to the public nature of blogs, this identity-work is projected onto a much larger stage than that described in other works. The “sacred space” that the bloggers craft for themselves ensures that their legacy or “ethical capital” potentially has far greater reach than those who do not project their thoughts and experiences about end-of-life into the public domain.

Blogging is thus an avenue for the development of empowering identity-work, which was not available in a pre-social media era. All three bloggers stated that they had a clear mission not only to share their experience with others but also to make a difference. Through blogging to mainly unidentified anonymous publics they ensure that their self becomes a public legacy. Blogs generally welcome and include a space for feedback from readers. This feedback has not been analyzed in this paper but the great majority of feedback in all the end-of-life blogs I read is positive generally praising the bloggers for sharing their stories, noting how helpful it is for others undergoing an end-of-life journey and providing messages of solidarity and/or support. This creates a positive feedback loop or a virtuous circle whereby writing a blog projects one’s authority and secures one’s legacy thus reinforcing self-identity, leading to positive feedback which encourages further blogging, empowerment and one’s identity of having a terminal diagnosis. Social media can thus be a powerful tool in the construction of identity.

### Implications for End-of-Life Care

Whilst there are major gaps and inequities in provision of palliative care (Dixon et al., 2015), providing appropriate care for people approaching the end-of-life to enhance their well-being and autonomy is a rising priority for the public, patients, carers and policy makers in the Anglo-Saxon western world (Carr and Luth, 2019). Sand et al. (2008) recommend allowing patients to take active part in their own care to preserve their sense of autonomy. The rhetoric of patient empowerment and choice is a feature of end-of-life policy documents (Department of Health, 2015), although there is recognition that patients will not always be able to realize their choices, e.g. If they would like hospice care but a bed is not available (see Borgstrom, 2015; Bailey et al., 2020).

How does the analysis of blogs which locates identity as key to combatting powerlessness inform debates about choice and end-of-life care? The bloggers all demonstrate a desire to determine their treatment and how to live but beyond this, they also use the blogs to construct an identity as a dying person. The blogs enable the bloggers to step back and reflect and thus create a space for personal change. Susan talks about a desire “to rise above the physical to a spiritual space” (Susan ‘On more time 19.8.2017) and Sophie talks about needing to enter a “sacred space.” Blogs are used to help develop this space and to communicate with others to reflect and test out one’s response to a terminal diagnosis in order to achieve personal growth. It is worth noting here that by no means everyone who receives a terminal diagnosis desires “choice” across the board. Many people in that position, for example, find themselves more comfortable ceding clinical (and other) decision-making to those around them. Moreover, only a very small minority choose to blog about the processes of dying. However, this analysis would suggest that interventions which focus on creating the space for the individual to explore who they are, how they can best be maintained to be themselves, will help to counteract powerlessness and enhance end-of-life care.

Prioritizing identity throws a different perspective on much of the literature and policy relating to end-of-life care as it goes beyond operationalizing patient choice as focusing on physical comfort, and the place and medicalization of death. It would suggest that end-of-life care should also attend to supporting the person to be who they want to be and perhaps, as others have suggested, place more emphasis on spiritual care, an aspect that is currently infrequent in end-of-life care (Balboni et al., 2013). Hospice care often incorporates this element offering a range of services (e.g. spiritual support; health and beauty) that would not be defined as healthcare. End-of-life care would be enriched by broadening its scope to routinely include the offer of such support.

### Reflections on Limitations

Whilst all three bloggers selected for this analysis demonstrate and re-affirm the power to be themselves when faced with a terminal diagnosis, they are by no means representative of the wider population. Bloggers are not typical as most people do not write a regular blog particularly about private issues such as illness and death. Medical bloggers tend to be well-educated, reasonably affluent and come from higher income countries (Kovic et al., 2008; Miller and Pole, 2010). This was the profile of the 32 bloggers I identified from which the three included here are drawn. They were all authored by those with the high levels of social, economic and cultural capital as identified by Bourdieu (Pinxten and Lievens, 2014). Blogging is rarely available to people living without access to a smart phone, the internet or an education. In this sense, blogs or access to social media more broadly serve to heighten inequalities in death.

The analysis was based on only three bloggers, two based in Canada and one in the UK and therefore the results may not be applicable to people living in different cultural or medical circumstances or contexts. Whereas the themes were reflected in the wider set of 32 blogs I identified, these were all written from bloggers based in the UK, North America or Australasia and it is likely that issues of identity and control are more salient in the Anglo-Saxon western world than other contexts. Furthermore, despite the homogeneity of those selected there were still marked contextual differences such as legality of medical assistance in dying, access to hospice care etc. In addition, the three selected were all aged late 40s or 50s whereas 14 of the 32 blogs I identified were written by people under the age of 40. The sense of loss of missed life opportunities and concern for young children who they were leaving behind was more pronounced among the younger bloggers.

Those who blog about having a terminal illness have by default acknowledged that their lifespan is limited. This is by no means a universal response to a terminal diagnosis, an issue I had a close encounter with when my sister was diagnosed with stage 4 cancer while I was in the middle of analyzing the blogs. Whilst she was open about her diagnosis and happy to talk to family, friends and colleagues about her treatment, she chose to ignore the word ‘palliative’ in letters from her oncologist and as her health deteriorated was initially reluctant to seek end-of-life care in a hospice. Rather, her focus was on living as normal and full a life as was possible continuing to work full-time, go on holidays, spend time with family and friends and buy new clothes. That was her response to powerlessness in the face of a terminal diagnosis and like the bloggers, demonstrated both agency and self-affirmation, but in a different way.

There is also evidence from the literature that people approaching end-of-life who do not blog may express the “power to be themselves.” Carlander et al. (2011) note that identity-work is a feature of the narratives they collated from people close to death. There are many examples of legacy projects such as videos, letters, poems, artwork or even collections of favourite recipes that people nearing the end-of-life collate to pass down to others (Allen et al., 2008). A study conducted in the United States including 1462 seriously ill and racially and socio-economically diverse patients, recently bereaved family, physicians and other health providers found that achieving a sense of completion was considered to be of key importance at the end-of-life (Steinhauser et al., 2000). Identity is also found to be a key element in end-of-life care in marginalized populations. A systematic review of Gypsy, Traveller and Roma experiences and needs in end-of-life care (Dixon et al., 2021) found the traveller identity to be a key concern expressed in strong family and community values which include a preference for healthcare to be provided from within the community and distinct health beliefs regarding superstitions around illness, personal care, death rituals and bereavement. Thus, while the expression and construction of a dying identity may vary in different cultural settings and contexts, the “power to be oneself” seems to be a concept that has wide applicability.

## Conclusion

Blogging is just one response selected by a minority of people with a terminal diagnosis. Despite their lack of representativeness of the wider terminally ill population, their blogs nevertheless provide insight into how social media is used to promote the “power to be oneself” and challenge the powerlessness often associated with end-of-life. This demonstrates the role patient empowerment plays in constructing a dying identity and how social media is used as a mechanism to promote this.

There are many ways in which people respond to a terminal diagnosis and different paths that people take to address the powerlessness that is sometimes associated with it. The challenge for people facing a terminal diagnosis as well as their significant others and carers is to determine the best path for them. This analysis of three blogs would suggest that in trying to identify the best path, the notion of maximizing the “power to be oneself” may be a good start.

## Data Availability

Publicly available datasets were analyzed in this study. The names of, and links to, the blogs are: 1) The death project available at: https://susanbriscoe.wordpress.com/ 2) Don’s journey available at: https://www.dyingwithdignity.ca/don_journey7 3) The cancer whisperer available at: https://www.cancerwhispering.com/blog.
